# Highly Active and Isospecific Styrene Polymerization Catalyzed by Zirconium Complexes Bearing Aryl-substituted [OSSO]-Type Bis(phenolate) Ligands

**DOI:** 10.3390/polym8020031

**Published:** 2016-01-26

**Authors:** Norio Nakata, Tomoyuki Toda, Yusuke Saito, Takanori Watanabe, Akihiko Ishii

**Affiliations:** Department of Chemistry, Graduate School of Science and Engineering, Saitama University, 255 Shimo-okubo, Sakura-ku, Saitama 338-8570, Japan; t.toda@mst.nagaokaut.ac.jp (T.T.); s13ds003@mail.saitama-u.ac.jp (Y.S.); s14mc129@mail.saitama-u.ac.jp (T.W.)

**Keywords:** zirconium, post-metallocene, styrene polymerization, [OSSO]-type ligand, isotactic

## Abstract

[OSSO]-type dibenzyl zirconium(IV) complexes **9** and **10** possessing aryl substituents ortho to the phenoxide moieties (ortho substituents, phenyl and 2,6-dimethylphenyl (Dmp)) were synthesized and characterized. Upon activation with dMAO (dried methylaluminoxane), complex **9** was found to promote highly isospecific styrene polymerizations ([*mm*] = 97.5%–99%) with high molecular weights *M*_w_ up to 181,000 g·mmol^−1^. When the Dmp-substituted pre-catalyst **10**/dMAO system was used, the highest activity, over 7700 g·mmol(**10**)^−1^·h^−1^, was recorded involving the formation of precisely isospecific polystyrenes of [*mm*] more than 99%.

## 1. Introduction

Stereo-controlled polystyrene such as syndiotactic (sPS) and isotactic polystyrenes (iPS) is one of the most versatile polymeric materials, owing to its high melting point, high crystallinity, and excellent resistance to heat and chemicals [1,2]. Since the syndiospecific polymerization of styrene catalyzed by homogeneous CpTiCl_3_/MAO system was discovered by Ishihara and coworkers at Idemitsu Kosan (Tokyo, Japan) [3,4], numerous efforts have been devoted to employ efficient metal-based catalysts for the production of sPS [5,6,7,8,9,10,11,12,13,14,15,16,17,18,19]. In sharp contrast, isotactic-enriched polystyrenes are still prepared using heterogeneous catalysis [20,21,22,23,24,25,26] or anionic polymerization [27,28,29,30]. While many homogeneous nickel catalysts for the synthesis of iPS have been reported [31,32,33], there are only a few examples of metallocene and post-metallocene catalysts that produce completely isotactic polystyrene [34,35]. Okuda *et al.* reported that MAO-activated titanium complex **1a** supported by 1,4-dithiabutane-bridged [OSSO]-type bis(phenolate) ligand catalyzes the styrene polymerization with good activity (330 g·mmol(**1a**)^−1^·h^−1^) to yield ultra-high molecular weight isotactic polystyrene (*M*_w_ = 5,300,000 g·mol^−1^) [36,37]. Very recently, they also demonstrated that thermally stable robust zirconium complex **2** possessing a dicumyl-substituted [OSSO]-type bis(phenolate) ligand can efficiently polymerize styrene in living fashion giving highly isotactic poly(styrene) ([*mm*] > 99%) with quite high activity up to 3158 g·mmol(**2**)^−1^·h^−1^ [38]. Capacchione and Proto *et al.* also presented the living isospecific polymerization of styrene and 1,3-dienes promoted by using [OSSO]-type titanium complex **1b** and MAO to form isotactic-poly(styrene)-*block*-poly(1,3-diene) copolymes [39,40]. *ansa*-Bridged bis(indenyl) allyl yttrium and neodymium complexes **3** developed by Carpentier *et al.* also acted as single-site catalysts for the completely isospecific polymerization of styrene with relatively high activity (**3a**: 1066–1637 g·mmol(**3a**)^−1^·h^−1^; **3b**: 392–1094 g·mmol(**3b**)^−1^·h^−1^) [41,42].

**Scheme 1:**
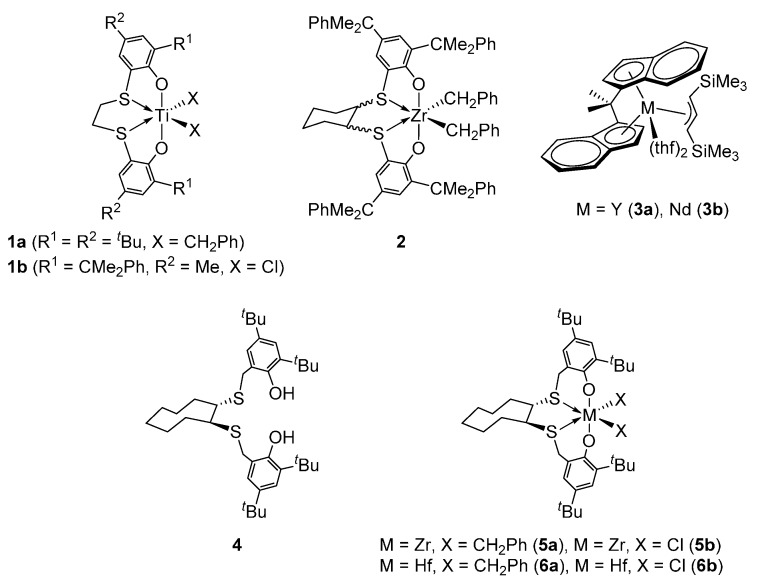
Related post-metallocene complexes **1**–**3** for styrene polymerization, [OSSO]-type ligand **4** and its complexes **5** and **6**.

Recently, we have succeeded in the development of an [OSSO]-type bis(phenolate) ligand (**4**) based on a *trans*-1,2-cyclooctanediyl platform and the preparation of several early-transition metal and aluminum complexes [43,44,45,46,47,48]. We have also found that zirconium(IV) and hafnium(IV) complexes **5** [49,50] and **6** [51] incorporating ligand **4** with activator could promote precisely isospecific polymerizations of α-olefins such as 1-hexene, 4-methyl-1-pentene, and propylene involving excellent activity (Scheme 1). However, the polymerization of styrene using complexes **5** or **6** and activator did not occur, even at high temperatures, probably due to steric hindrance by bulky *t*Bu groups at the ortho positions on the phenolate moieties in **4**. These results encouraged us to develop a new type of [OSSO]-type bis(phenolate) ligands, which have a suitable coordination environment to achieve the isospecific styrene polymerization. Herein, we present the synthesis and structural characterization of [OSSO]-type dibenzyl zirconium(IV) complexes possessing aryl substituents ortho to the phenoxide moieties, as well as their ability to catalyze isospecific styrene polymerization.

## 2. Experimental Section

### 2.1. General

All manipulations of air- and/or moisture-sensitive compounds were performed either using standard Schlenk-line techniques or in UNICO 650F (Ibaraki, Japan) or Glovebox Japan E300 gloveboxes (Tokyo, Japan) under an inert atmosphere of argon. Hexane and toluene were purchased from Kanto Chemical (Tokyo, Japan) and were dried over a potassium mirror prior to use. C_6_D_6_ was dried over a potassium mirror, and it was degassed by a freeze–thaw cycle prior to use. ^1^H and ^13^C NMR spectra were recorded on a Bruker DPX-400 (400 and 101 MHz, respectively, (Billerica, MA, USA) using CDCl_3_ or C_6_D_6_ as the solvent at room temperature. High-resolution mass spectrometry (HRMS) data were recorded by using a Hitachi-Hitec NanoFrontier eLD (Tokyo, Japan). All melting points were determined on a Mel-Temp capillary tube apparatus (Saitama, Japan) and are uncorrected. The molecular weights (*M*_w_) and molecular weight distributions (*M*_w_/*M*_n_) of the polymers were evaluated by gel permeation chromatography (GPC) on a SCL-10AVP/LC-10ATVP/DGU-14A/ CTO-10ACVP/RID-10A apparatus (Shimadzu Corporation, Kyoto, Japan) using a GPC KF-804L (Shodex Corporation, Tokyo, Japan) column. The analyses were performed at room temperature using THF as the solvent and standard polystyrene as the reference. Differential scanning calorimetry (DSC) analyses were performed on a Seiko Instruments DSC 6200 apparatus (Tokyo, Japan) at a rate of 5 °C/min, under continuous flow of N_2_ (0.5 mL/min), using aluminum capsules. [OSSO]-type ancillary ligands **7** and **8** [53] and dMAO [54] were prepared by the literature procedures.

### 2.2. Preparation of Dibenzyl Zirconium(IV) Complex **9**

A solution of **7** (298 mg, 0.430 mmol) in toluene (10 mL) was added to a solution of Zr(CH_2_Ph)_4_ [55] (196 mg, 0.430 mmol) in toluene (10 mL) at room temperature. The mixture was stirred for 1 h at room temperature, and the solvent was removed under reduced pressure. The residue was washed with hexane and dried *in vacuo* to give dibenzyl zirconium(IV) complex **9** (346 mg) in 83% yield as yellow crystals. **9**: Mp 274–275 °C (dec.).

^1^H NMR (400 MHz) δ 0.65 (br s, 2H), 0.91 (br s, 2H), 1.07 (br s, 6H), 1.29–1.41 (m, 6H), 1.38 (d, *J* = 9 Hz, 2H), 2.01 (d, *J* = 9 Hz, 2H), 2.39 (br s, 2H), 3.15 (d, *J* = 14 Hz, 2H), 3.35 (d, *J* = 14 Hz, 2H), 6.50 (d, *J* = 7 Hz, 4H), 6.88 (d, *J* = 2 Hz, 2H), 6.96–7.21 (m, 14 H), 7.34–7.40 (m, 8H), 7.54 (d, *J* = 2 Hz, 2H), 7.67 (d, *J* = 7 Hz, 4H).

^13^C{^1^H} NMR (101 MHz) δ 25.4 (*C*H_2_), 26.1 (*C*H_2_), 28.7 (*C*H_2_), 34.5 (*C*H_2_), 48.5 (*C*H), 59.3 (*C*H_2_), 123.1 (*C*), 123.3 (*C*H), 127.0 (2*C*H), 127.6 (*C*H), 128.6 (*C*H), 128.7 (*C*H), 129.1 (2*C*H), 129.2 (2*C*H), 129.3 (2*C*H), 129.9 (2*C*H), 130.5 (*C*H), 130.8 (2*C*H), 132.5 (*C*), 133.3 (*C*), 140.1 (*C*), 141.1 (*C*), 144.4 (*C*), 158.2 (C).

### 2.3. Preparation of Dibenzyl Zirconium(IV) Complex **10**

A solution of **8** (380 mg, 0.608 mmol) in toluene (10 mL) was added to a solution of Zr(CH_2_Ph)_4_ [55] (277 mg, 0.608 mmol) in toluene (5 mL) at room temperature. The mixture was stirred for 1 h at room temperature, and the solvent was removed under reduced pressure. The residue was washed with hexane (2 mL) and dried to give dibenzyl zirconium(IV) complex **10** (499 mg, 92%) as yellow crystals. **10**: Mp 240–241 °C (dec.).

^1^H NMR (400 MHz, C_6_D_6_) δ 0.76 (m, 2H), 0.82 (d, *J* = 8 Hz, 2H), 1.00 (m, 2H), 1.17–1.30 (m, 6H), 1.45–1.55 (m, 4H), 1.65 (d, *J* = 8 Hz, 2H), 2.05 (s, 6H), 2.10 (s, 1H), 2.26 (s, 6H), 2.38 (s, 6H), 2.41 (br s, 1H), 3.04 (d, *J* = 15 Hz, 2H), 3.11 (d, *J* = 15 Hz, 2H), 6.33 (br s, 2H), 6.47 (d, *J* = 7 Hz, 4H), 6.71 (br s, 2H), 6.91 (t, *J* = 7 Hz, 2H), 7.07 (t, *J* = 7 Hz, 4H), 7.11–7.15 (m, 4H), 7.25 (d, *J* = 7 Hz, 2H).

^13^C{^1^H} NMR (101 MHz, C_6_D_6_) δ 20.6 (*C*H_3_), 21.3 (*C*H_3_), 21.4 (*C*H_3_), 21.9 (*C*H_2_), 25.4 (*C*H_2_), 26.2 (*C*H_2_), 34.3 (*C*H_2_), 48.1 (*C*H), 58.0 (*C*H_2_), 122.0 (*C*), 122.8 (*C*H), 127.6 (*C*H), 127.8 (*C*H), 127.9 (*C*H), 129.2 (2*C*H), 129.3 (*C*), 129.6 (2*C*H), 130.2 (*C*H), 130.8 (*C*), 131.4 (*C*H), 136.2 (*C*), 137.4 (*C*), 139.9 (*C*), 144.6 (*C*), 156.1 (*C*).

### 2.4. General Procedure for Styrene Polymerization

A 50 mL Schlenk-flask was charged sequentially with catalytic precursor **9** or **10** (2.0 μmol), dMAO as an activator (0.50 mmol), and toluene (5 mL) at 25 °C. After stirring for 5 min at the temperature, styrene (3.0 g, 28.8 mmol) was added to the reaction mixture. The mixture was stirred for 60, 10, or 5 min at a desired temperature. The reaction was quenched by addition of methanol and HCl aq. The mixture was extracted with CH_2_Cl_2_ and the organic layer was washed with water and dried over anhydrous Na_2_SO_4_. The solvent was removed *in vacuo* at 70 °C during overnight to leave poly(styrene).

### 2.5. X-ray Crystallographic Analysis

Yellow single crystals of **9** were grown by slow evaporation of its saturated hexane solution at −20 °C. The intensity data were collected at 100 K for **9** on a Bruker SMART APEX II ULTRA (Billerica, MA, USA) equipped with a CCD area detector with graphite-monochromated MoKa radiation (*l* = 0.71073 Å). The structure was solved by direct methods and refined by full-matrix least-squares procedures on *F*^2^ for all reflections (SHELX-97) [56]. Hydrogen atoms of **9** were located by assuming ideal geometry and were included in the structure calculations without further refinement of the parameters. Crystallographic data and details of refinement for **9**: C_60_H_56_O_2_S_2_Zr, 2(C_7_H_8_), *M*_w_ = 1148.66, orthorhombic, space group *P*2_1_2_1_2_1_, *a* = 11.8318(11) Å, *b* = 21.988(2) Å, *c* = 22.933(2) Å, *V* = 5966.1(10) Å^3^, *Z* = 4, *D*_calc_ = 1.279 g cm^−3^, *R*_1_ (*I* > *2*σ*I*) = 0.0434, *wR*_2_ (all data) = 0.1053 for 11081 reflections, 495 restraints, and 833 parameters, GOF = 1.017.

## 3. Results and Discussion

### 3.1. Synthesis of Dibenzyl Zirconium(IV) Complexes **9** and **10**

**Scheme 2:**
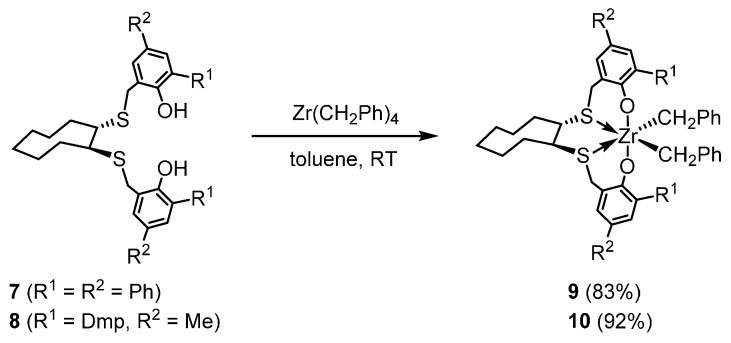
Synthesis of aryl-substituted [OSSO]-type dibenzyl zirconium(IV) complexes **9** and **10**.

According to a previous report [53], new [OSSO]-type ancillary ligands **7** and **8** with *ortho*, *para*-diphenylphenol or *ortho*-2,6-dimethylphenyl (Dmp), *para*-methylphenol substituents, respectively, were prepared in the total yields of 45% or 39%, respectively. Treatment of [OSSO]-type ligands **7** or **8** with Zr(CH_2_Ph)_4_ in toluene at room temperature gave the corresponding dibenzyl zirconium(IV) complexes **9** or **10** as air- and moisture-sensitive pale yellow crystals in 83% or 92% yields, respectively (Scheme 2). Similarly to the NMR observation of the related [OSSO]-type dibenzyl zirconium(IV) complex **5** [49,50], all NMR data of **9** and **10** showed the magnetical equivalency of two phenolate moieties as well as the two benzyl ligands, indicating that complexes **9** and **10** take a *C*_2_-symmetric, helical structure on the NMR time scale. For example, in the ^1^H NMR, AB patterns due to the *S*-benzyl protons appeared at δ 3.15 and 3.34 with *J* = 14 Hz for **9** and at δ 3.04 and 3.11 with *J* = 15 Hz for **10**. The sulfur-bonded methine protons in the cyclooctane ring were observed at δ 2.39 for **9** and δ 2.41 for **10** as a broad singlet.

**Figure 1:**
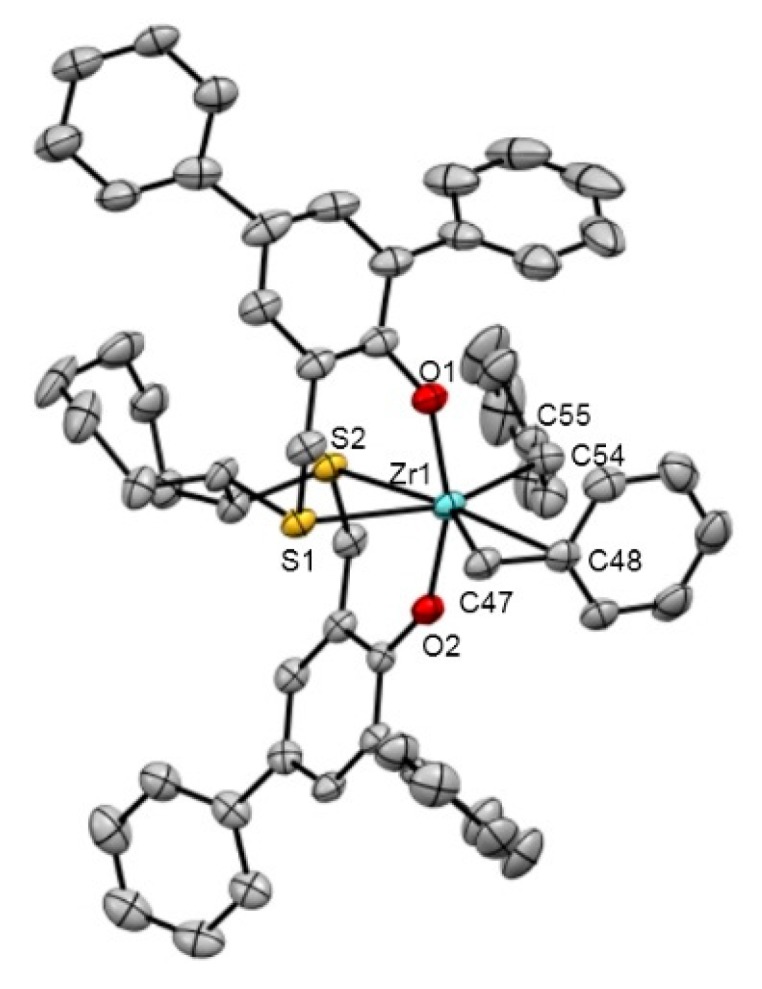
ORTEP drawing of dibenzyl zirconium(IV) complex **9** (50% thermal ellipsoids, hydrogen atoms and solvated toluene molecules were omitted for clarity). Selected bond lengths Å and bond angles °: Zr1–O1 = 1.998 (2), Zr1–O2 = 2.005 (2), Zr1–C47 = 2.305 (3), Zr1–C48 = 2.592 (3), Zr1–C54 = 2.321 (4), Zr1–S1 = 2.8148 (11), Zr1–S2 = 2.8079 (9), O1–Zr1–O2 = 160.25 (9), S1–Zr1–S2 = 70.74 (3), C47–Zr1–C54 = 121.54 (13), Zr1–C47–C48 = 84.4 (2), Zr1–C54–C55 = 124.3 (3), S1–C1–C2–S2 = 67.3 (3).

The molecular structure of diphenyl derivative **9** was confirmed by X-ray crystallography, as shown in Figure 1. In the crystalline state, the zirconium center possesses a distorted octahedral geometry with *cis*-arranged two sulfur atoms and two benzyl groups, adopting a *cis*-α configuration as similar as the previously reported [OSSO]-type zirconium(IV) complexes [49,50,57,58,59,60]. One benzyl ligand of **9** is coordinated to the zirconium center by η^1^-mode with the Zr–C_benzyl_–C_ipso_ angle of 124.3(2)°, whereas the other has an acute Zr–C_benzyl_–C_ipso_ angle of 84.4(2)° consistent with the η^2^-coordination fashion as similarly as in the case of *t*Bu-substituted complex **5a**; the Zr-C_ipso_ distance (2.592(3) Å) is extremely shortened compared with that in **5a** (2.820(3) Å) [48], indicating that this η^2^-benzyl group in **9** is strongly bound to the metal than that in **5a** due to the less steric hindrance around the zirconium center in **9** as expected. The Zr–S bond lengths in **9** (2.8073(7), 2.8148(11) Å) are comparable to those in **5a** (2.8107(8), 2.7682(8) Å) [49,50] and [Zr{2,2′-(OC_6_H_2_-4,6-Br_2_)_2_ CH_2_SCH_2_CH_2_SCH_2_}(CH_2_Ph)_2_] (2.7934(7), 2.7932(6) Å) [61].

### 3.2. Styrene Polymerization

**Figure 2:**
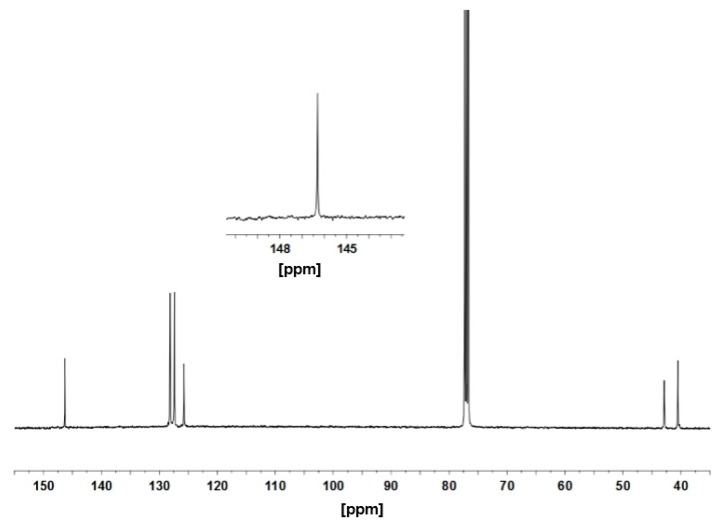
^13^C{^1^H} NMR spectrum of polystyrene obtained by the **9**/dMAO system at 0 °C (Table 1, Run 1).

To elucidate the potential as an isospecific polymerization catalyst, we examined the coordinate polymerization of styrene using precursors **9** and **10**. The results at varied temperatures are compiled in Table 1. The polymerization of styrene (3.0 g, 28.8 mmol) with **9** (2.0 μmol) upon activation with 250 equiv. of dMAO (500 μmol) in toluene at 0 °C proceeded slowly to yield a crystalline polystyrene (0.099 g) (Run 1). The microstructure of the polystyrene was determined by ^13^C NMR spectroscopy, which showed six sharp signals to mean an excellent isotacticity over 99% of the [*mm*] triad (Figure 2). Since no stereo error was observed around the lowest resonance at δ 146.3 assigned to the phenyl *ipso* carbon, the detail microstructure is attributed to the [*mm*] heptad. Similarly to the case for *ansa*-type complexes **3** reported by Carpentier [41,42], an enantiomorphic site control mechanism is operating for the stereospecific propagation in our system, probably due to the racemic coordination of the [OSSO]-type ligand framework. This high isotactic microstructure can be corroborated from the melting temperature (*T*_m_ = 218.6 °C) determined by DSC analysis. The GPC analysis of the resulting polymer revealed a large molecular weight (*M*_w_ = 137,000 g·mol^−1^) and a monomodal distribution with a very broad polydispersity (PDI = 8.6). When the polymerization temperature was raised to 25 or 40 °C, slightly decreasing isotacticity of 96.8% or 90.7% together with narrower molecular weight distributions of 3.8 or 2.9 were observed, respectively (Runs 2 and 3). At 70 °C, **9**/dMAO system showed the highest activity of 618 g·mmol(**9**)^−1^·h^−1^, and the narrow PDI value of 2.2, while the isotacticity of the obtained polymer decreased somewhat to 87.5% and the molecular weight *M*_w_ was reduced drastically to 9500 g·mol^−1^, suggesting that the active species was unstable during the chain propagation step at high temperatures (Run 4). In the varying temperature experiments, the broader polydispersity and the higher molecular weight *M*_w_ were observed at lower temperatures as characteristic trends. These results would be explained by a relatively slow generation of active species and/or a slow propagation of polymer chains in the styrene polymerization process.

We then turned our attention to the use of Dmp-substituted complex **10**. The polymerization of styrene (3.0 g, 28.8 mmol) employing the system consisting of **10** (2.0 μmol) and dMAO (500 μmol) in toluene at 0 °C for 10 min could also afford completely isotactic polystyrene of 0.259 g ([*mm*] > 99%), which corresponds to the activity of 777 g·mmol(**10**)^−1^·h^−1^ (Run 5). Despite the more overcrowded environment at zirconium center in **10**, this activity is approximately 15 times higher than that of **9** at the same temperature (50 g·mmol(**9**)^−1^·h^−1^). GPC analysis exhibited that the obtained polymer was monomodal and had a high molecular weight (*M*_w_ = 257,000 g·mol^−1^) and a slightly large polydispersity of 3.1. Similar results were observed in the polymerizations carried out at 25 and 40 °C to produce excellent isotactic polymers ([*mm*] > 99%) with higher molecular weights *M*_w_ up to 380,000 g·mol^−1^ and narrower molecular weight distributions of 1.8 and 2.1, respectively, being consistent with a single site behavior (Runs 6 and 7). The corresponding activities recorded in the range of 2200–4100 g·mmol(**10**)^−1^·h^−1^ increased with elevating temperatures; at 70 °C, **10**/dMAO system achieved the highest polymerization activity of 7700 g·mmol(**10**)^−1^·h^−1^ forming a high molecular weight polystyrene (*M*_w_ = 195,000 g·mol^−1^, Run 8). Despite the higher polymerization temperature, the resulting polymer had a complete isotacticity ([*mm*] > 99%) and a monomodal molecular weight distribution (*M*_w_/*M*_n_ = 1.8). In the DSC measurement, the melting temperatures (*T*_m_ = 221.4–225.8 °C) of polymers produced by **10**/dMAO system reflected their highly isotactic microstructures. Thus, our catalyst system exhibited remarkably higher activity, even at low temperatures (777–7700 g·mmol(**10**)^−1^·h^−1^ at 0–70 °C) than those of reported [OSSO]-type titanium complex **1a** (330 g·mmol(**1**)^−1^·h^−1^ at 40 °C) [8] and *ansa*-type yttrium and neodymium complexes **3a** (1066–1637 g·mmol(cat)^−1^·h^−1^ at 80–120 °C) and **3b** (392–1094 g·mmol(cat)^−1^·h^−1^ at 60–100 °C) [11].

**Table 1:** Styrene polymerization with dibenzyl zirconium(IV) complexes **9** and **10** upon activation with dMAO.

Run	Cat.	Temp.(°C)	Time (min)	Activity (g·mmol^−1^·h^−1^)	*M*_w_ (g·mol^−1^)	PDI *^b^*	(*mm*) *^c^* (%)	*T*_m_ *^d^* (°C)
1	**9**	0	60	50	137,000	8.6	>99	218.6
2	**9**	25	60	139	156,000	3.8	96.8	209.8
3	**9**	40	60	182	181,000	2.9	90.7	-
4	**9**	70	60	618	9500	2.2	87.5	-
5	**10**	0	10	777	257,000	3.1	>99	225.3
6	**10**	25	10	2200	380,000	2.1	>99	225.8
7	**10**	40	5	4100	338,000	1.8	>99	222.6
8	**10**	70	5	7700	195,000	1.8	>99	221.4

*^a^* Conditions: **9** and **10** 2.0 μmol, [dMAO]/[Cat.] = 250, styrene 3.0 g (28.8 mmol), toluene 5 mL; *^b^* PDI = *M*_w_/*M*_n_, determined by GPC (PS standard); *^c^* Determined by ^13^C{^1^H} NMR spectrum *^d^* Determined by DSC.

## 4. Conclusions

We have established a controlled isospecific polymerization of styrene employing dibenzyl zirconium(IV) complexes (**9** and **10**) supported by new aryl-substituted [OSSO]-type bis(phenolate) ligands based on *trans*-cyclooctanediyl platform. Use of the Dmp-substituted pre-catalyst **10** in the presence of dMAO as an activator was critical for the formation of precisely isospecific polystyrenes with high activities. Notably, the isotactic polymers produced by **10**/dMAO system had significantly larger molecular weights and narrower polydispersity indexes of nearly 2.0.

## Supplementary Materials

Supplementary materials can be accessed at www.mdpi.com/2073-4360/8/2/31/s1. Figure S1. ^1^H-NMR spectrum of dibenzyl zirconium(IV) complex **9**; Figure S2. ^1^H-NMR spectrum of dibenzyl zirconium(IV) complex **10**; Figure S3. ^13^C{^1^H}-NMR spectrum of polystyrene obtained by the 10/dMAO system at 0 °C (Table 1, Run 5); Figure S4. ^13^C{^1^H}-NMR spectrum of polystyrene obtained by the 10/dMAO system at 25 °C (Table 1, Run 6); Figure S5. ^13^C{^1^H}-NMR spectrum of polystyrene obtained by the 10/dMAO system at 40 °C (Table 1, Run 7); Figure S6. ^13^C{^1^H}-NMR spectrum of polystyrene obtained by the 10/dMAO system at 70 °C (Table 1, Run 8); Figure S7. DSC chart of polystyrene obtained by the 10/dMAO system at 0 °C (Table 1, Run 5); Figure S8. DSC chart of polystyrene obtained by the 10/dMAO system at 25 °C (Table 1, Run 6); Figure S9. DSC chart of polystyrene obtained by the 10/dMAO system at 40 °C (Table 1, Run 7); Figure S10. DSC chart of polystyrene obtained by the 10/dMAO system at 70 °C (Table 1, Run 8); Scheme S1. Preparation of dibenzyl zirconium(IV) complex **9**; Scheme S2. Preparation of dibenzyl zirconium(IV) complex 10.
