# Supplementary material for: Highly Active and Isospecific Styrene Polymerization Catalyzed by Zirconium Complexes Bearing Aryl-substituted [OSSO]-Type Bis(phenolate) Ligands
Source: Polymers (Basel). 2016 Jan 26;8(2):31. doi: 10.3390/polym8020031 (PMC6432521; doi:10.3390/polym8020031)
Supplement: Supplementary file 1 [file polymers-08-00031-s001.pdf]

Supplementary Materials: Highly Active and Isospecific Styrene Polymerization Catalyzed by Zirconium Complexes Bearing Aryl-substituted [OSSO]-Type Bis(phenolate) Ligands

Norio Nakata, Tomoyuki Toda, Yusuke Saito, Takanori Watanabe and Akihiko Ishii

Table of Contents

NO.	Contents
1	Figure S1. ^{1}H-NMR spectrum of dibenzyl zirconium(IV) complex **9**.
2	Figure S2. ^{1}H-NMR spectrum of dibenzyl zirconium(IV) complex **10**.
3	Figure S3. ^{13}C{^{1}H}-NMR spectrum of polystyrene obtained by the 10/dMAO system at 0 °C (Table 1, Run 5).
4	Figure S4. ^{13}C{^{1}H}-NMR spectrum of polystyrene obtained by the 10/dMAO system at 25 °C (Table 1, Run 6).
5	Figure S5. ^{13}C{^{1}H}-NMR spectrum of polystyrene obtained by the 10/dMAO system at 40 °C (Table 1, Run 7).
6	Figure S6. ^{13}C{^{1}H}-NMR spectrum of polystyrene obtained by the 10/dMAO system at 70 °C (Table 1, Run 8).
7	Figure S7. DSC chart of polystyrene obtained by the 10/dMAO system at 0 °C (Table 1, Run 5).
8	Figure S8. DSC chart of polystyrene obtained by the 10/dMAO system at 25 °C (Table 1, Run 6).
9	Figure S9. DSC chart of polystyrene obtained by the 10/dMAO system at 40 °C (Table 1, Run 7).
10	Figure S10. DSC chart of polystyrene obtained by the 10/dMAO system at 70 °C (Table 1, Run 8).

Experimental Section

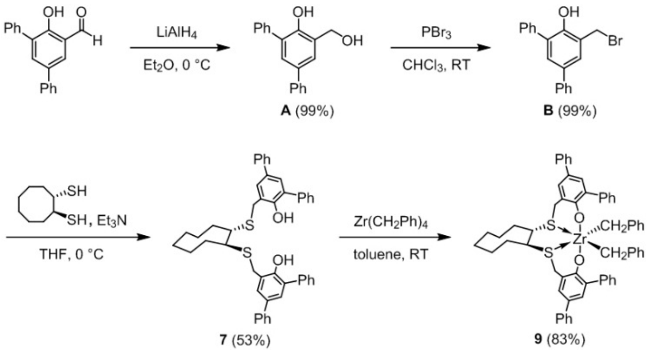

Scheme S1. Preparation of dibenzyl zirconium(IV) complex **9**.

Preparation of (2-hydroxy-3,5-diphenyl)benzyl alcohol (A)

To a solution of 3,5-diphenylsalicylaldehyde [1] (830 mg, 3.03 mmol) in Et_2O (20 mL) was added $LiAlH_4$ (115 mg, 3.03 mmol) at 0 °C. The mixture was stirred at 0 °C for 2 h and then quenched with H_2O and HCl aq. and diluted with Et_2O. The organic layer was separated, washed with water, and dried over anhydrous Na_2SO_4. After the removal of the solvent in vacuo, almost pure (2-hydroxy-3,5-diphenyl)benzyl alcohol **A** (834 mg,) was obtained in 99% yield as a colorless oil.

A: ^{1}H-NMR (400 MHz, $CDCl_3$) δ 2.52 (s, 1H), 4.88 (s, 2H), 6.90 (s, 1H), 7.23–7.47 (m, 12H).

^{13}C{^{1}H}-NMR (101 MHz, $CDCl_3$) δ 64.2 (CH_2), 126.4 (*C*), 126.5 (CH), 126.9 (2CH), 127.1 (CH), 127.9 (CH), 129.0 (2CH), 129.0 (2CH), 129.1 (CH), 129.5 (2CH), 129.6 (*C*), 133.7 (*C*), 137.5 (*C*), 140.7 (*C*), 151.9 (*C*). HR-MS (ESI, *m*/*z*) calcd. for $C_{19}H_{16}O_2Na^+$ 299.10425; found 299.10342 $[M]^+$.

Preparation of (2-hydroxy-3,5-diphenyl)benzyl bromide (B)

To a solution of benzyl alcohol A (834 mg, 3.02 mmol) in $CHCl_3$ (20 mL) was gradually added PBr_3 (276 mg, 1.02 mmol) at room temperature. The mixture was stirred at room temperature for 1 h and then quenched with H_2O. The organic layer was separated, washed with water, and dried over anhydrous Na_2SO_4. After the removal of the solvent in vacuo, almost pure (2-hydroxy-3,5-diphenyl)benzyl bromide **B** (1.02 g) was obtained in 99% yield as a colorless oil.

B: ^{1}H-NMR (400 MHz, $CDCl_3$) δ 4.68 (s, 2H), 7.23 (s, 1H), 7.31–7.49 (m, 12H).

^{13}C{^{1}H}-NMR (101 MHz, $CDCl_3$) δ 29.4 (CH_2), 125.0 (*C*), 126.9 (2*C*H), 127.2 (*C*H), 128.6 (*C*H),128.9(*C*) 128.9 (2*C*H), 129.2 (*C*H), 129.3 (2*C*H), 129.6 (*C*H), 129.7 (2*C*H), 134.2 (*C*), 136.5 (*C*), 140.2 (*C*), 150.4 (*C*).

Preparation of *trans*-1,2-bis[(2-hydroxy-3,5-diphenylbenzyl)sulfanyl]cyclooctane (7)

To a mixture of *trans*-cyclooctane-1,2-dithiol [2] (268 mg, 1.52 mmol) and benzyl bromide B (1.030 g, 3.04 mmol) in THF (20 mL) was added Et_3N (310 mg, 3.04 mmol) at 0 °C. The mixture was stirred at ambient temperature for 2 days, and the solvent was removed under reduced pressure. The reaction mixture was extracted with Et_2O, and the organic layer was washed with NH_4Cl aq. and H_2O, and dried over anhydrous Na_2SO_4. After the removal of the solvent in vacuo, the residue was subjected to column chromatography (SiO_2, CH_2Cl_2/hexane = 1/1) to give bis(phenol) **7** (533 mg) in 53% yield as a colorless oil.

7: ^{1}H-NMR (400 MHz) δ 1.32–1.43 (m, 4H), 1.51–1.53 (m, 2H), 1.72–1.74 (m, 2H), 1.84–1.88 (m, 2H), 2.10–2.15 (m, 2H), 2.98–2.99 (m, 2H), 3.93 (d, *J* = 13 Hz, 2H), 3.98 (d, *J* = 13 Hz, 2H), 6.48 (s, 2H), 7.25–7.44 (m, 24H).

^{13}C{^{1}H}-NMR (101 MHz) δ 26.1 (CH_2), 26.1 (CH_2), 31.0 (CH_2), 33.4 (CH), 50.8 (CH_2), 124.2 (*C*), 126.9 (2CH), 127.0 (CH), 127.7 (CH), 128.7 (CH), 128.8 (2CH), 128.8(CH), 128.9 (2CH), 129.5 (2CH), 130.2 (*C*), 133.7 (*C*), 137.8 (*C*), 140.6 (*C*), 151.5 (*C*). HR-MS (ESI, *m*/*z*) calcd. for $C_{40}H_{48}O_2S_2Na^+$ 647.29880; found 647.30035 $[M]^+$.

Preparation of Dibenzyl Zirconium(IV) Complex 9

A solution of **7** (298 mg, 0.430 mmol) in toluene (10 mL) was added to a solution of $Zr(CH_2Ph)_4$ [3] (196 mg, 0.430 mmol) in toluene (10 mL) at room temperature. The mixture was stirred for 1 h at room temperature, and the solvent was removed under reduced pressure. The residue was washed with hexane and dried under vacuo to give dibenzyl zirconium(IV) complex **9** (346 mg) in 83% yield as yellow crystals. **9**: Mp 274–275 °C (dec.).

^{1}H-NMR (400 MHz) δ 0.65 (br s, 2H), 0.91 (br s, 2H), 1.07 (br s, 6H), 1.29–1.41 (m, 6H), 1.38 (d, *J* = 9 Hz, 2H), 2.01 (d, *J* = 9 Hz, 2H), 2.39 (br s, 2H), 3.15 (d, *J* = 14 Hz, 2H), 3.35 (d, *J* = 14 Hz, 2H), 6.50 (d, *J* = 7 Hz, 4H), 6.88 (d, *J* = 2 Hz, 2H), 6.96–7.21 (m, 14 H), 7.34–7.40 (m, 8H), 7.54 (d, *J* = 2 Hz, 2H), 7.67 (d, *J* = 7 Hz, 4H).

^{13}C{^{1}H}-NMR (101 MHz) δ 25.4 (CH_2), 26.1 (CH_2), 28.7 (CH_2), 34.5 (CH_2), 48.5 (CH), 59.3 (CH_2), 123.1 (C), 123.3 (CH), 127.0 (2CH), 127.6 (CH), 128.6 (CH), 128.7 (CH), 129.1 (2CH), 129.2 (2CH), 129.3 (2CH), 129.9 (2CH), 130.5 (CH), 130.8 (2CH), 132.5 (C), 133.3 (C), 140.1 (C), 141.1 (C), 144.4 (C), 158.2 (C).

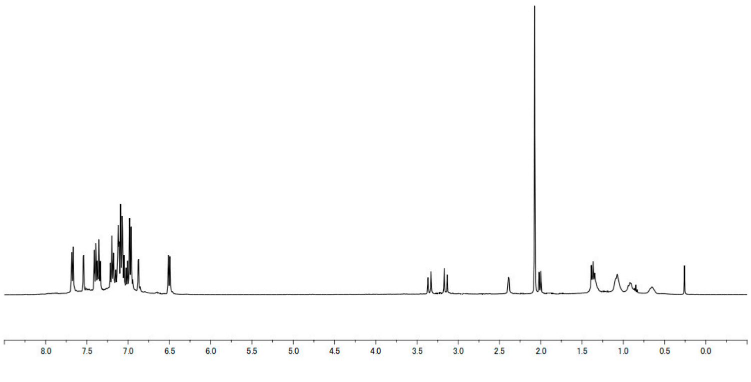

Figure S1. ^{1}H-NMR spectrum of dibenzyl zirconium(IV) complex 9.

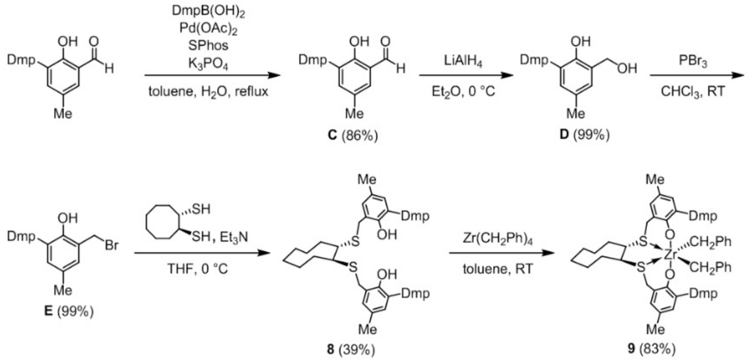

Scheme S2. Preparation of dibenzyl zirconium(IV) complex **10**.

Preparation of 3-(2,6-dimethylphenyl)-5-methylsalicylaldehyde (C)

To a mixture of 3-bromo-5-methylsalicylaldehyde [4] (442 mg, 1.87 mmol), 2,6-dimethylphenylboronic acid (368 mg, 2.45 mmol), $Pd(OAc)_2$ (14 mg, 0.062 mmol), SPhos (2-dicyclohexylphosphino-2′,6′-dimethoxybiphenyl) (50 mg, 0.122 mmol), and K_3PO_4 (1.30 g, 6.1 mmol) in toluene (15 mL) and water (3 mL) was refluxed for 1 d. The solvent was removed under reduced pressure, and the residue was diluted with Et_2O. The organic layer was separated, washed with water, dried over anhydrous Na_2SO_4. After the removal of the solvent in vacuo, the residue was subjected to column chromatography (SiO_2, Et_2O) to give 3-(2,6-dimethylphenyl)-5-methylsalicylaldehyde **C** (385 mg) in 86% yield as a colorless oil.

C: ^{1}H-NMR (400 MHz, $CDCl_3$) δ 2.10 (s, 6H), 2.42 (s, 3H), 7.16–7.20 (m, 5H), 7.36–7.37 (m, 1H), 9.94 (s, 1H), 11.01 (s, 1H).

^{13}C{^{1}H}-NMR (101 MHz, $CDCl_3$) δ 20.2 (CH_3), 20.3 (2CH_3), 120.5 (*C*), 127.2 (2*C*H), 127.6 (*C*H), 129.0 (*C*), 129.5 (*C*), 132.7 (*C*H), 135.8 (*C*), 136.5 (2*C*), 139.1 (*C*H), 156.5 (*C*), 196.6 (*C*H). HR-MS (ESI, *m*/*z*) calcd. for $C_{16}H_{16}O_2Na^+$ 263.10425; found 263.10440 $[M]^+$.

Preparation of [2-hydroxy-3-(2,6-dimethylphenyl)-5-methyl]benzyl alcohol (D)

To a solution of 3-(2,6-dimethylphenyl)-5-methylsalicylaldehyde **C** (385 mg, 1.60 mmol) in Et_2O (15 mL) was added $LiAlH_4$ (121 mg, 3.20 mmol) at 0 °C. The mixture was stirred at 0 °C for 10 min, and then quenched with H_2O and HCl aq. and diluted with Et_2O. The organic layer was separated, washed with water, dried over anhydrous Na_2SO_4. After the removal of the solvent in vacuo, almost pure [2-hydroxy-3-(2,6-dimethylphenyl)-5-methyl]benzyl alcohol **D** (387 mg) was obtained in 99% yield as a colorless oil.

D: ^{1}H-NMR (400 MHz, $CDCl_3$) δ 2.05 (s, 6H), 2.30 (s, 3H), 4.78 (s, 2H), 5.44 (br s, 1H), 6.79 (d, *J* = 2 Hz, 1H), 7.02 (d, *J* = 2 Hz, 1H), 7.14–7.23 (m, 3H).

^{13}C{^{1}H}-NMR (101 MHz, $CDCl_3$) δ 20.5 (CH_3), 20.6 (2CH_3), 63.0 (CH_2), 126.1 (*C*), 127.3 (*C*), 127.7 (2*C*H), 128.0 (*C*H), 128.4 (*C*H), 129.4 (*C*), 130.0 (*C*H), 135.9 (*C*), 137.6 (2*C*), 149.1 (*C*). HR-MS (ESI, *m*/*z*) calcd. for 265.11990; found 265.11980 $[M]^+$.

Preparation of [2-hydroxy-3-(2,6-dimethylphenyl)-5-methyl]benzyl bromide (E)

To a solution of benzyl alcohol D (387 mg, 1.60 mmol) in $CHCl_3$ (20 mL) was added PBr_3 (0.1 mL, 1.05 mmol) at room temperature. The mixture was stirred at room temperature for 1 h and then quenched with H_2O. The organic layer was separated, washed with water, dried over anhydrous Na_2SO_4. After the removal of the solvent in vacuo, almost pure (2-hydroxy-3,5-diphenyl)benzyl bromide **E** (511 mg) was obtained in 99% yield as a colorless oil.

E: ^{1}H-NMR (400 MHz, $CDCl_3$) δ 2.04 (s, 6H), 2.29 (s, 3H), 4.60 (s, 2H), 6.01 (br, 1H), 6.80 (d, *J* = 1.6 Hz, 1H), 7.13–7.24 (m, 4H).

^{13}C{^{1}H}-NMR (101 MHz, $CDCl_3$) δ 20.5 (CH_3), 20.6 (2CH_3), 29.5 (CH_2), 124.0 (*C*), 126.9 (*C*), 128.0 (2*C*H), 128.6 (*C*H), 130.1 (*C*H), 130.7 (*C*), 130.1 (*C*H), 134.4 (*C*), 138.0 (2*C*), 148.5 (*C*).

Preparation of *trans*-1,2-bis{[2-hydroxy-3-(2,6-dimethylphenyl)-5-methylbenzyl]-sulfanyl} cyclooctane (8).

To a mixture of *trans*-cyclooctane-1,2-dithiol (140 mg, 0.798 mmol) and benzyl bromide E (511 mg, 1.68 mmol) in THF (20 mL) was added Et_3N (365 mg, 3.60 mmol) at 0 °C. The mixture was stirred at room temperature for 15 h and the solvent was removed under reduced pressure. The reaction mixture was extracted with Et_2O, and the organic layer was washed with NH_4Cl aq. and H_2O, and dried over anhydrous Na_2SO_4. After the removal of the solvent in vacuo, the residue was subjected to column chromatography (SiO_2, CH_2Cl_2/hexane = 1/1) to give bis(phenol) **8** (196 mg) in 39% yield as a colorless oil.

8: ^{1}H-NMR (400 MHz, $CDCl_3$) δ 1.26 (br s, 2H), 1.32–1.43 (m, 2H), 1.32–1.43 (m, 2H), 1.50–1.53 (m, 4H), 1.69–1.81 (m, 4H), 2.02 (s, 6H), 2.03 (s, 6H), 2.25 (s, 6H), 2.95–2.97 (m, 2H), 3.83 (d, *J* = 13 Hz, 2H), 3.88 (d, *J* = 13 Hz, 2H), 5.48 (s, 2H), 6.73 (d, *J* = 2 Hz, 2H), 7.03 (d, *J* = 2 Hz, 2H), 7.08–7.16 (m, 6H).

$^{13}C\{^1H\}$-NMR (101 MHz) δ 20.5 (CH_3), 20.7 (CH_3), 20.5 (CH_3), 25.9 (CH_2), 26.2 (CH_2), 30.6 (CH_2), 32.6 (CH), 50.5 (CH_2), 123.7 (*C*), 127.7 (*C*), 127.9 (2CH), 129.7 (CH), 129.8 (CH), 130.6 (*C*), 136.3(CH), 137.5 (*C*), 137.6 (2*C*), 149.1 (CH). HR-MS (ESI, *m*/*z*): calcd. for $C_{46}H_{44}O_2S_2Na^+$ 715.26750; Found 715.26701 $[M]^+$.

Preparation of dibenzyl zirconium(IV) Complex 10

A solution of **8** (380 mg, 0.608 mmol) in toluene (10 mL) was added to a solution of $Zr(CH_2Ph)_4$ (277 mg, 0.608 mmol) in toluene (5 mL) at room temperature. The mixture was stirred for 1 h at room temperature, and the solvent was removed under reduced pressure. The residue was washed with hexane (2 mL) and dried to give dibenzyl zirconium(IV) complex **10** (499 mg, 92%) as yellow crystals. **10**: Mp 240–241 °C (dec.).

^{1}H-NMR (400 MHz, C_6D_6) δ 0.76 (m, 2H), 0.82 (d, *J* = 8 Hz, 2H), 1.00 (m, 2H), 1.17–1.30 (m, 6H), 1.45–1.55 (m, 4H), 1.65 (d, *J* = 8 Hz, 2H), 2.05 (s, 6H), 2.10 (s, 1H), 2.26 (s, 6H), 2.38 (s, 6H), 2.41 (br s, 1H), 3.04 (d, *J* = 15 Hz, 2H), 3.11 (d, *J* = 15 Hz, 2H), 6.33 (br s, 2H), 6.47 (d, *J* = 7 Hz, 4H), 6.71 (br s, 2H), 6.91 (t, *J* = 7 Hz, 2H), 7.07 (t, *J* = 7 Hz, 4H), 7.11–7.15 (m, 4H), 7.25 (d, *J* = 7 Hz, 2H).

$^{13}C\{^1H\}$-NMR (101 MHz, C_6D_6) δ 20.6 (CH_3), 21.3 (CH_3), 21.4 (CH_3), 21.9 (CH_2), 25.4 (CH_2), 26.2 (CH_2), 34.3 (CH_2), 48.1 (CH), 58.0 (CH_2), 122.0 (*C*), 122.8 (CH), 127.6 (CH), 127.8 (CH), , 127.9 (CH), 129.2 (2CH), 129.3 (*C*), 129.6 (2CH), 130.2 (CH), 130.8 (*C*), 131.4 (CH), 136.2 (*C*), 137.4 (*C*), 139.9 (*C*), 144.6 (*C*), 156.1 (*C*).

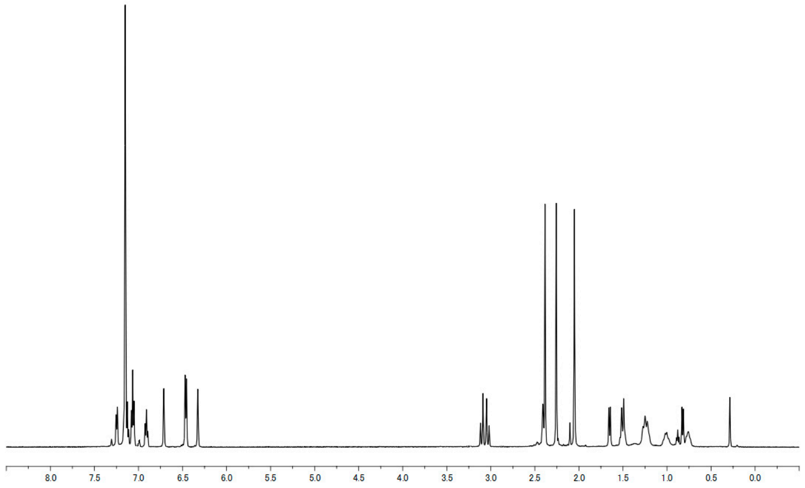

Figure S2. ^{1}H-NMR spectrum of dibenzyl zirconium(IV) complex **10**.

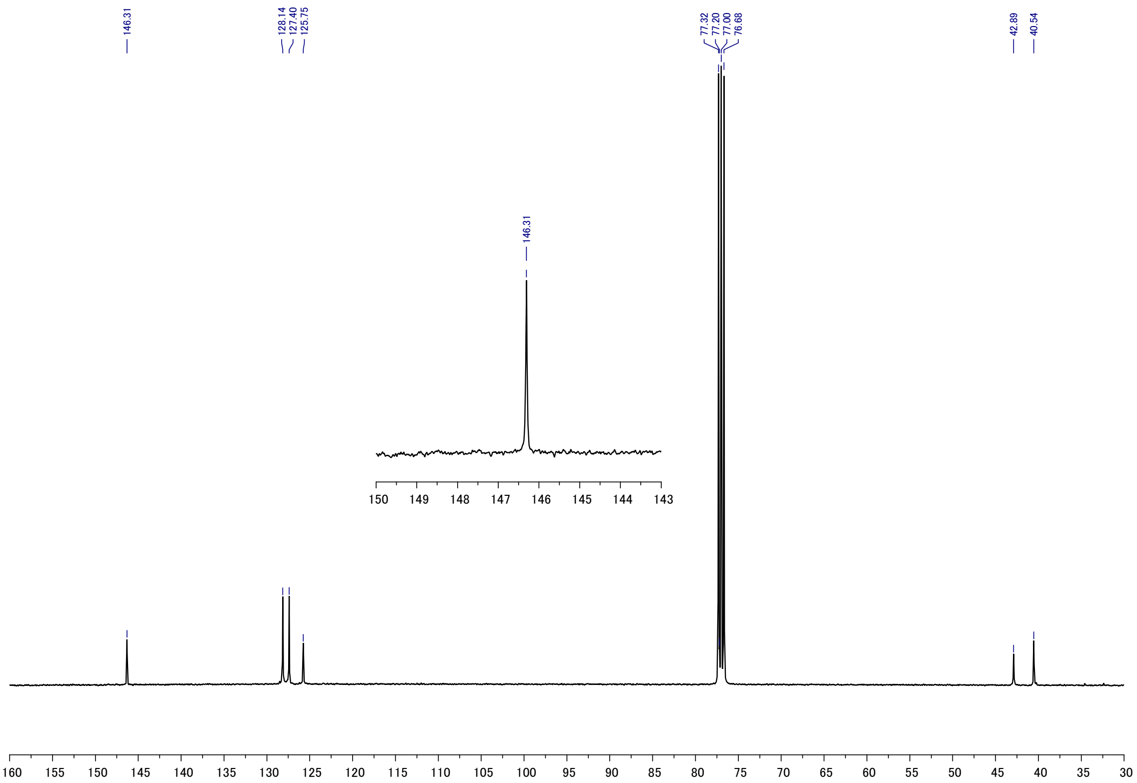

Figure S3. $^{13}C\{^1H\}$-NMR spectrum of iPS obtained by the **10**/dMAO system at 0 °C (Table 1, Run 5).

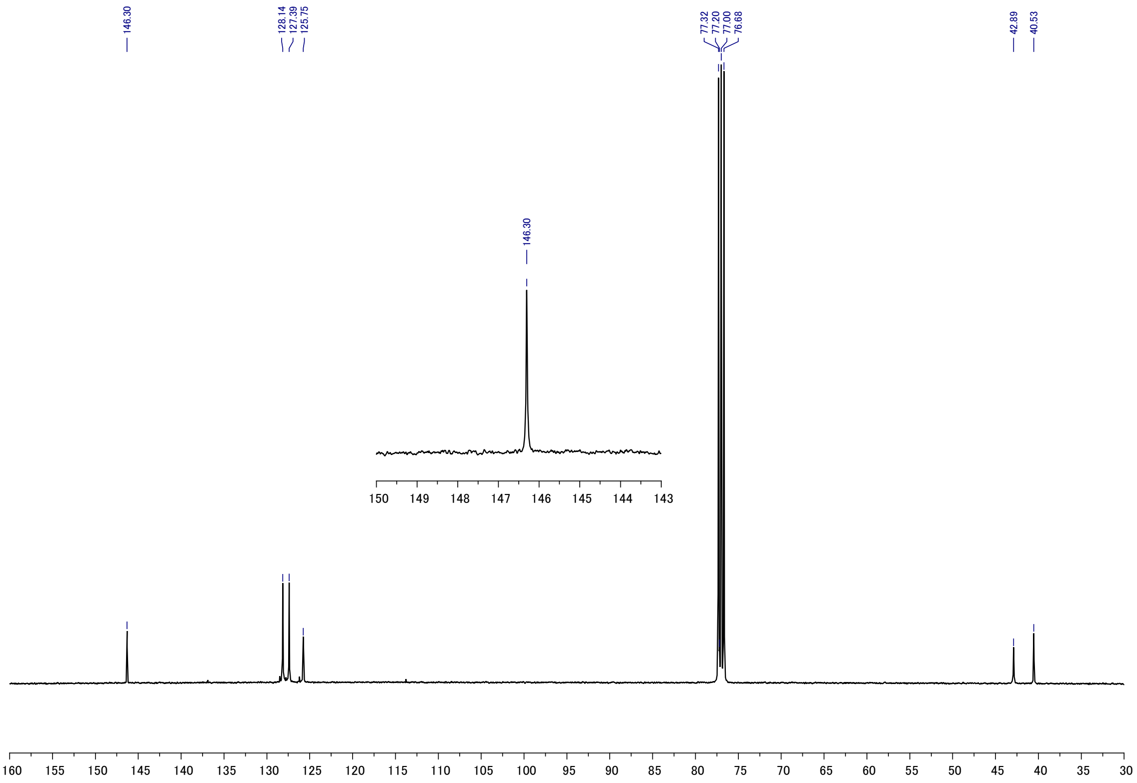

Figure S4. $^{13}C\{^1H\}$-NMR spectrum of iPS obtained by the **10**/dMAO system at 25 °C (Table 1, Run 6).

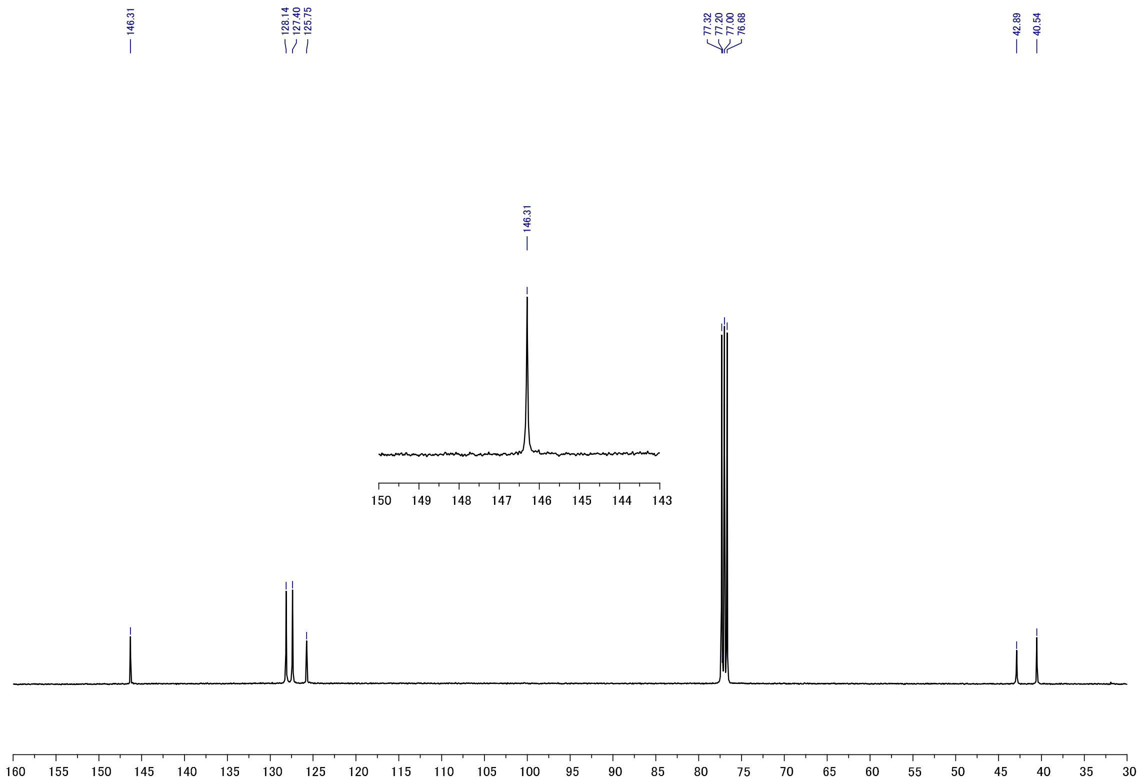

Figure S5. $^{13}C\{^1H\}$-NMR spectrum of iPS obtained by the **10**/dMAO system at 40 °C (Table 1, Run 7).

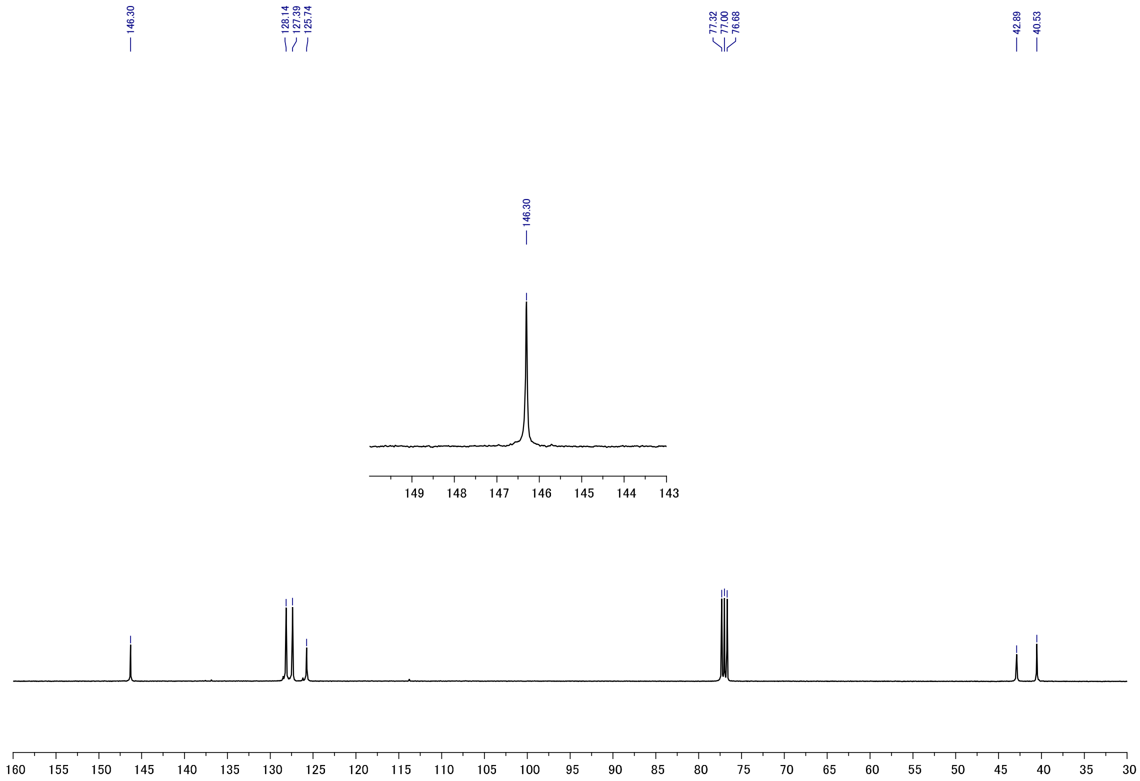

Figure S6. $^{13}C\{^1H\}$-NMR spectrum of iPS obtained by the **10**/dMAO system at 70 °C (Table 1, Run 8).

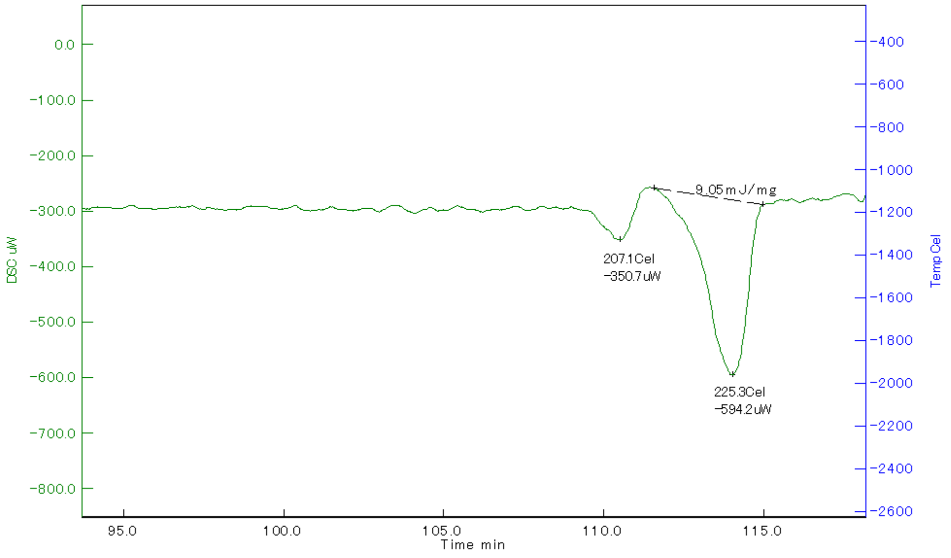

Figure S7. DSC thermogram of iPS obtained by the **10**/dMAO system at 0 °C (Table 1, Run 5).

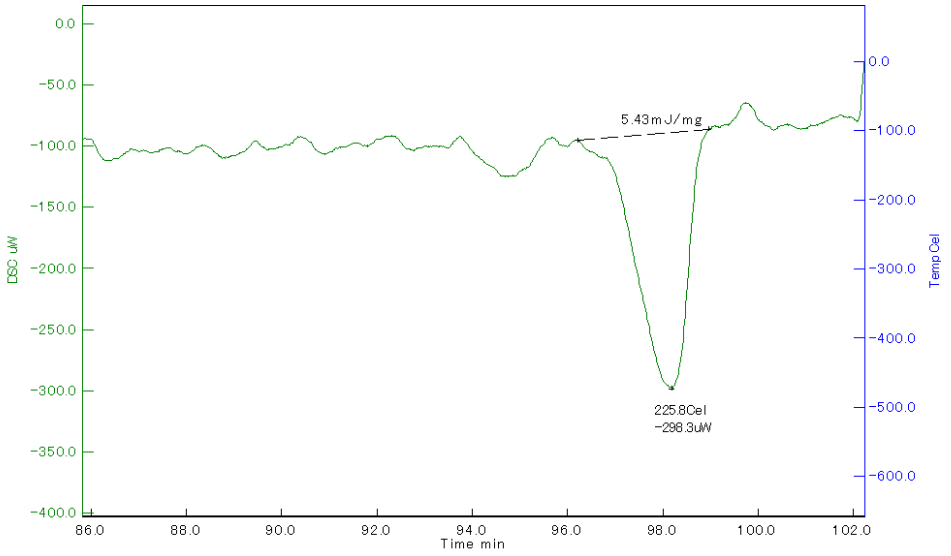

Figure S8. DSC thermogram of iPS obtained by the **10**/dMAO system at 25 °C (Table 1, Run 6).

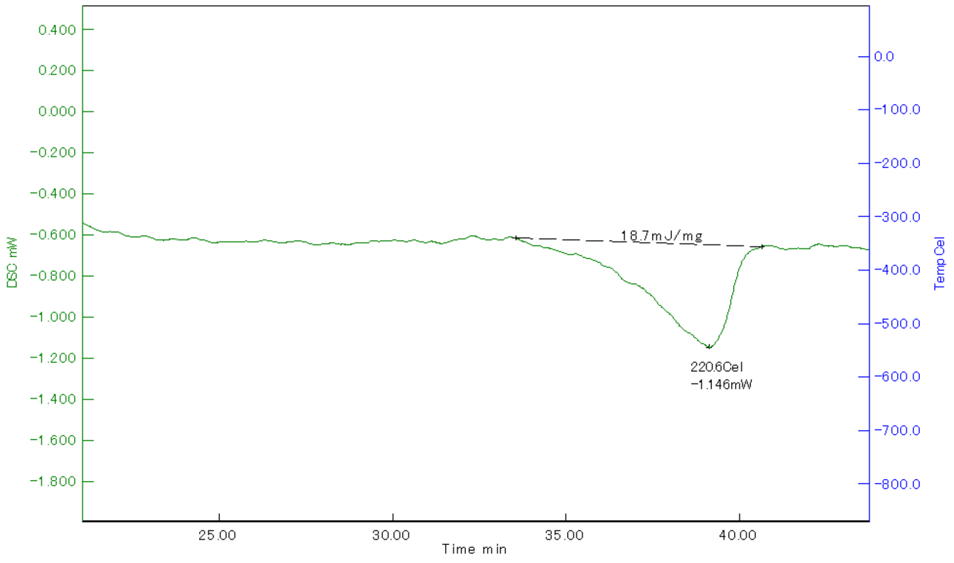

Figure S9. DSC thermogram of iPS obtained by the **10**/dMAO system at 40 °C (Table 1, Run 7).

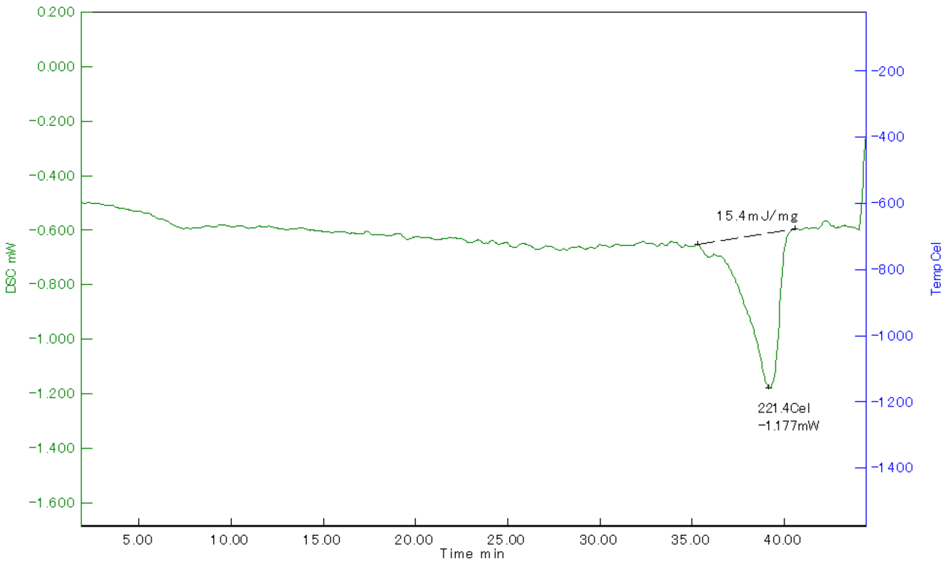

Figure S10. DSC thermogram of iPS obtained by the **10**/dMAO system at 70 °C (Table 1, Run 8).

General Procedure for Styrene Polymerization

A 50 mL Schlenk-flask was charged sequentially with catalytic precursor 9 or 10 (2.0 mol), dMAO as an activator (0.50 mmol), and toluene (5 mL) at a desired temperature. After stirring for 1 min at the temperature, styrene (3.0 g, 28.8 mmol) was added to the reaction mixture. The mixture was stirred for 60, 10, or 5 min at the temperature. The reaction was quenched by addition of methanol and HCl aq. The mixture was extracted with CH_2Cl_2 and the organic layer was washed with water and dried over anhydrous Na_2SO_4. The solvent was removed in vacuo at 70 °C during overnight to leave poly(styrene).

References

1. Zhuravel, M.A.; Nguyen, S.T. Preparation of 3-aryl-substituted salicylaldehydes via Suzuki coupling. *Tetrahedron Lett.* **2001**, *42*, 7925–7928.
2. Ishii, A.; Ono, A.; Nakata, N. Three syntheses of *trans*-cyclooctane-1,2-dithiol by ring opening of *cis*-cyclooctene episulfoxide with ammonium thiocyanate followed by reduction and reductions of *trans*-1,2-di(thiocyanato)cyclooctane and *trans*-1,2-cyclooctyl trithiocarbonate. *J. Sulfur Chem.* **2009**, *30*, 236−244.
3. Rong, Y.; Al-Harbi, A.; Parkin, G. Highly variable Zr–CH_2–Ph bond angles in tetrabenzylzirconium: analysis of benzyl ligand coordination modes. *Organometallics* **2012**, *31*, 8208−8217.
4. Verner, E.; Katz, B.A.; Spencer, J.R.; Allen, D.; Hataye, J.; Hruzewicz, W.; Hui, H.C.; Kolesnikov, A.; Li, Y.; Luong, C.; *et al*. Development of serine protease inhibitors displaying a multicentered short (<2.3 Å) hydrogen bond binding mode: Inhibitors of urokinase-type plasminogen activator and factor Xa. *J. Med. Chem.* **2001**, *44*, 2753–2771.

© 2016 by the authors; licensee MDPI, Basel, Switzerland. This article is an open access article distributed under the terms and conditions of the Creative Commons by Attribution (CC-BY) license (http://creativecommons.org/licenses/by/4.0/).
